# Exploring Brain Size Asymmetry and Its Relationship with Predation Risk Among Chinese Anurans

**DOI:** 10.3390/biology14010038

**Published:** 2025-01-07

**Authors:** Chuan Chen, Ying Jiang, Yiming Wu, Lingsen Cao, Wenbo Liao

**Affiliations:** 1 Key Laboratory of Southwest China Wildlife Resources Conservation (Ministry of Education), China West Normal University, Nanchong 637009, China; chenchuan@bjfu.edu.cn (C.C.); jiangyingjuly@163.com (Y.J.); yiming.wu@cwnu.edu.cn (Y.W.); ls_11242024@163.com (L.C.); 2 School of Ecology and Nature Conservation, Beijing Forestry University, Beijing 100083, China; 3 Key Laboratory of Artificial Propagation and Utilization in Anurans of Nanchong City, China West Normal University, Nanchong 637009, China; 4 College of Panda, China West Normal University, Nanchong 637009, China

**Keywords:** asymmetry, anurans, brain region, environmental factor, predation risk

## Abstract

Brain size asymmetry differs considerably across species, including humans, vertebrates, and invertebrates. The subtle structural, functional, or size differences between the two brain sides are associated with processing specific cognitive tasks. To evaluate the differences between the sizes of the left and right sides of the whole brain and brain regions and the effect of predation risk (i.e., snake density) on brain size asymmetry among Chinese anurans, we compared the differences between the left and right hemisphere sizes of the whole brain and brain regions among anuran species and analyzed the correlations between the predation risk and size asymmetry index of the brain and brain regions. We found that when one side of the brain was consistently larger than the other, there was a significant difference between the sizes of the left and right sides of the brain and brain regions, displaying directional asymmetry of the whole brain and brain regions. We also found that total brain size was positively correlated with the size asymmetry index of the olfactory bulb and optic tecta when the left hemispheres of the whole brain and brain regions were larger than the right ones. Meanwhile, the index of telencephalon size asymmetry was positively correlated with predation risk when the right hemispheres of the brain and brain regions were larger than the left ones. However, there were non-significant differences between the sizes of the left and right sides of the brain and brain regions across 99 species of anurans. Our findings suggest that an increased predation risk linked to sociality is likely to drive an increase in right telencephalon size.

## Simple Summary

Brain size asymmetry has been implicated in specific cognitive tasks in a broad range of animals. This study aimed to ascertain the asymmetry of brain size among Chinese anurans and the effect of predation risk (i.e., snake density) on the index of brain size asymmetry. The results showed that a significant difference was observed between the sizes of the left and right parts of the brain and brain regions, displaying directional size asymmetry of the total brain and brain regions when one side is consistently larger than the other. For all 99 species of anurans, there was a non-significant difference between the left and right hemisphere size of the whole brain and brain regions.

## 1. Introduction

Biologists have confirmed that a fundamental property of biological systems is their asymmetrical organization during the evolutionary process [1,2]. Symmetry, such as bilateral symmetry, is often associated with harmony and a form of predictable rigidity [3]. The degree of symmetry in bilateral organ structures is used as an indicator of changes in environmental conditions [4]. Bilateral symmetry, also known as “fluctuating asymmetry (FA)”, sometimes reflects developmental stability in organisms [5]. Fluctuating asymmetry is often considered an indicator of the genome’s ability to successfully buffer development and achieve a normal phenotype under stressful environments [6]. Conversely, directional asymmetry is another type of bilateral asymmetry in which one side is consistently larger than the other [7,8] that is also seen as a potential measure of developmental stability [9]. There is evidence that the degree of directional asymmetry in the organ size of animals is associated with developmental stability [10,11,12].

Comparative analyses of brain size evolution have primarily focused on the relationships between brain size and ecological and life history aspects in organisms [13,14,15,16,17,18,19,20,21,22,23,24,25,26,27,28,29,30,31,32,33,34,35]. The human brain exhibits a considerable number of morphological and functional left–right differences associated with environmental factors [36,37,38]. For example, the degree of functional lateralization is positively associated with the level of cognitive ability in humans [37,38]. Similarly, in non-human vertebrates and lower animals, normal variation and specialization produce asymmetry in the whole brain and brain structures [39,40,41]. The degree and direction of brain hemispheric asymmetry differ significantly among species [42]. Previous studies of the relationship between brain size and brain asymmetry have confirmed that larger-brained species tend to have more right-lateralized hemispheres in human subjects [43,44].

Brain asymmetry has been conserved or independently acquired during the evolutionary process, suggesting some cognitive advantage. For instance, many regional cortical asymmetries exist in chimpanzees, rhesus macaques, olive baboons, and vervet monkeys [45,46,47,48,49], indicating that brain asymmetry is likely associated with primate-specialized functions, such as communication and tool use [50]. The most dramatic illustration of the effects of environmental factors on brain asymmetry is light stimulation on the asymmetric development of the visual pathway in birds [51,52,53]. In particular, the bird embryos are turned in the egg so that light passing through the shell can only stimulate the right eye in the final days of development. Indeed, eye preference for avoiding predator risk is dependent on the early exposure of embryos to light in zebrafish [54]. Additionally, the effect of environmental temperature on sex hormones can explain gender differences observed in lateralized behaviors in fish [55]. There is evidence that sex hormones modulate seasonal variations in habenular structural asymmetry in frogs [56]. However, it remains unclear whether brain size asymmetry is associated with environmental factors (e.g., predator risk) across anuran species.

Anurans provide an opportunity to examine the relationship between brain size asymmetry and predation risk. Snakes are major predators of anurans in China [57]. Anuran species have developed various morphological and behavioral traits specialized for coping with different levels of predation risk [21,23,58,59]. If specialized behaviors associated with predators show evidence of brain lateralization in anurans, we would expect that detecting predators using a left visual hemifield should show a right hemisphere bias, thus displaying brain size asymmetry. Indeed, there is evidence for left eye–right hemisphere specialization for predator response in three species of toads [22]. However, there is no evidence that right hemispheric specialization for responding to predators is associated with asymmetry in the size of the hemispheres. Neural pathways and subcellular aspects of brain functions may differ between the hemispheres without being manifested as hemispheric differences in brain size. In this study, our aims were to investigate the effects of predator risk on the size asymmetry of the whole brain and brain regions among species of Chinese anurans. First, we tested whether there were left–right differences in the sizes of total brain and brain regions among these species. Second, if left–right differences in the sizes of the whole brain and brain regions existed for some species, we tested the relationships between the index of directional asymmetry of the brain and total brain size. Finally, we examined the relationships between the index of size asymmetry of the whole brain and brain regions and predator risk among anurans when one side was larger than the other.

## 2. Materials and Methods

### 2.1. Field Samples

**Figure 1:**
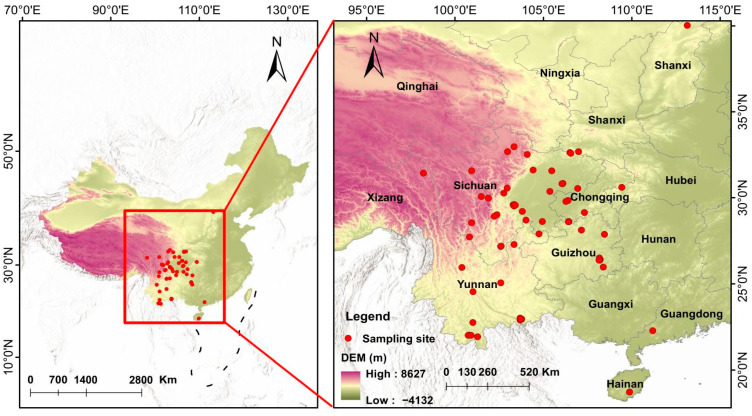
Topographic map showing the locations of the 99 species of anurans in China.

A total of 459 individuals from 99 Chinese anuran species were collected from April to July between 2017 and 2020 in western and southwestern China (Figure 1; Table S1). We used a 12 V flashlight to search for all mature individuals along an area with an average length of 1.3 km (range: 0.4–4.6 km) and a width of 5 m and then captured and counted all individuals in their breeding season. Two people walked the transect for 2–3 h during the sampling process. For some transects, we collected all individuals in breeding ponds along the sampling strip transect. A number of individuals of each species were caught in the sampling transect, as shown in Table S1. They were sexed on the basis of the nuptial pad in adult males and eggs readily visible through the skin of the abdomen in females. All individuals were taken to the laboratory and kept individually in rectangular tanks (0.5 m × 0.4 m × 0.4 m). We used benzocaine to anesthetize and euthanize them through single-pithing. We measured the body size (snout–vent length: SVL) of each individual to the nearest 0.01 mm with a caliper and preserved them in 4% phosphate-buffered formalin. After two months, we dissected the brains of all the preserved individuals. The reported experiments comply with the current laws of China concerning animal experimentation, and permission to collect and sacrifice individuals was received from the Ethical Committee for Animal Experiments of China West Normal University (CWNU2019D002).

### 2.2. Predation Risk

The level of predation risk was determined based on the snake density at breeding sites, focusing on one breeding population per species in the breeding season from April to July between 2017 and 2020. For three consecutive nights around the full moon, we walked along transects with an average length of 1.3 km (range: 0.4–4.6 km) and a width of 5 m for each anuran population with a 12 V flashlight and recorded the number of snakes encountered. Specifically, we used a snake clip to capture the snakes found in the transect for two to three hours and then photographed and identified their classification status (Table S1). Subsequently, we released them to the sites where they were captured. Most of the snake species identified were known predators of anurans, including *Cyclophiops major*, *Trimeresurus stejnegeri*, *Protobothrops mucrosquamatus*, *Rhabdophis tigrinus*, *Lycodon ruhstrati*, *Ovophis tonkinensis*, *Dinodon rufozonatum*, *Elaphe taeniura*, and *Gloydius strauchii*. Nightly searches for a month for one population for each of 10 anuran species showed that searching for three consecutive nights around the full moon was sufficiently representative. There is evidence that the mean snake density is strongly correlated between the two approaches [21]. Therefore, we used the density of snakes for three consecutive nights to estimate the predation risk for other anurans. Finally, we calculated the mean number of snake individuals per km² of transect area as an indicator of predation risk.

### 2.3. Brain Measurements

**Figure 2:**
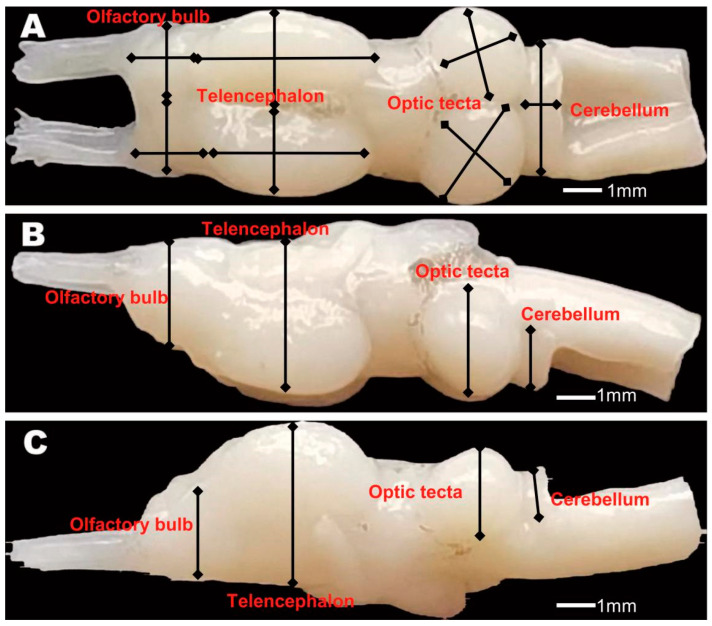
Ventral (**A**), right lateral (**B**), and left lateral (**C**) views of brain regions in an anuran species. Length, width, and height measures for each of the three main brain regions (olfactory bulb, telencephalon, and optic tectum) are shown.

All dissections and digital imaging were performed by two individuals (Yu JP and Mai CL). We obtained 459 intact brains of anurans in this study. Measurements were taken blindly by ID number without knowledge of species identity. We selected specific brain regions (olfactory bulbs, telencephalon, and optic tectum) for asymmetry analysis because they demonstrated significant differences between the left and right hemispheres. We obtained the left and right volumes of the whole brain and three main brain regions (i.e., olfactory bulbs, telencephalon, and optic tectum) for 99 anuran species (Table S2). The total brain size was calculated using the sum of the left and right volumes of the whole brain. Digital images of the dorsal, ventral, left, and right sides of the brain were captured using a Motic Images 3.1 digital camera mounted on a Moticam 2006 light microscope (Canon, Beijing, China) at 400× magnification. For dorsal and ventral views, we ensured that the brain was horizontally and symmetrically positioned so that neither hemisphere appeared larger than the other. For paired structures, we measured the length, width, and height of the left and right hemispheres using landmark-based measurements (tpsDig) version 1.37 software (Figure 2). The whole brain and brain regions were defined as the greatest distances enclosed by the landmarks used. Volumetric estimates of the whole brain and brain regions were based on the following ellipsoid model: volume = (L × W × H) × π/(6 × 1.43). Measurements were taken three times on random specimens to assess repeatability. We found very high intra-measurer repeatability within each species in the dataset (all R > 0.96). The potential subtle impacts of microevolution on brain size were minimized by sampling each species from a single locality [20]. The index of asymmetry for the total brain and brain regions was calculated using the following ellipsoid model: asymmetry = (left − right)/0.5 × (left + right) [60,61,62,63,64]. 

### 2.4. Phylogeny Reconstruction

To reconstruct the phylogeny of the 99 anuran species, we obtained sequences of six mitochondrial ribosomal genes and three nuclear genes from GenBank. The mitochondrial ribosomal genes included cytochrome b (CYTB), 12S ribosomal RNA (12S), 16S ribosomal RNA (16S), cytochrome oxidase I (COI), NADH dehydrogenase subunit 2 (ND2), and NADH dehydrogenase subunit 4 (ND4). The nuclear genes included recombination activating gene 1 (RAG1), tyrosinase (TYR), and ras homolog family member D (RHOD). We aligned the sequences using the multisequence alignment tool MUSCLE in MEGA v.6.0.6 [65]. The best nucleotide substitution model, which was determined using jModelTest v.2.1.7 [66] based on the Akaike information criterion, was GTR + Γ + I for all genes, except RHOD, for which HKY + Γ + I was favored. GTR + Γ + I was used as the best substitution model for all genes.

**Figure 3:**
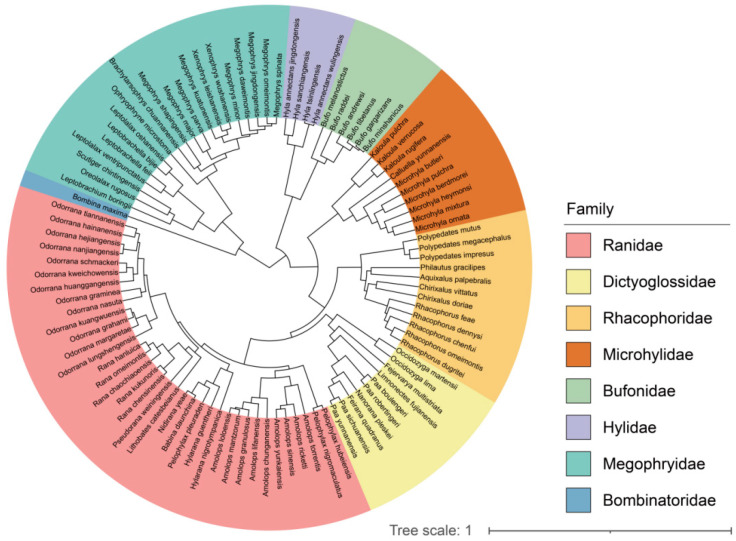
Phylogenetic tree of the 99 anurans species based on the three nuclear genes (RAG1, RHOD, and TYR) and the three mitochondrial genes (CYTB, 12S, and 16S), using TreeAnnotator v.1.8.3 in the comparative analysis.

To construct the phylogeny, we used unlinked substitution models, a relaxed uncorrelated log-normal clock, and a Yule speciation process with BEAUTi and BEAST v.1.8.3 [67]. Due to the lack of fossil dates for anurans, we omitted time calibration. We then used the BEAST implementation in the CIPRES Science Gateway (http://www.phylo.org) (accessed on 28 June 2024) to run the Markov Chain Monte Carlo (MCMC) simulation for 100 million generations, sampling every 10,000th tree. For all tree statistics, the effective sample size values exceed 200 in Tracer v.1.6.0 [68], indicating satisfactory convergence of the Bayesian chain and adequate model mixing. Finally, we used TreeAnnotator v.1.8.3 [67] to generate maximum clade credibility trees with mean node heights and a 10% burn-in (Figure 3). 

### 2.5. Statistical Analysis

All analyses were conducted on log_10_-transformed data in R version 4.0.1 [69]. We accounted for the non-independence of data due to shared ancestry using phylogenetic generalized least-squares (PGLS) models [70] based on the phylogeny described. To address varied sample sizes between species that might affect the accuracy of species means, we performed the PGLS analyses using the R package nlme, incorporating Pagel’s [71] phylogenetic correlation structure (corPagel), as implemented in the ape package [72], and weighting the model by the number of individuals measured. The PGLS models can estimate the phylogenetic scaling parameter λ based on maximum likelihood [73]. We assessed the phylogenetic effect by comparing our model with estimated λ to models with λ set to 0 (phylogenetic independence) or 1 (complete phylogenetic dependence) using likelihood ratio tests [70]. *p*-values for these tests were reported as superscripts following the λ values.

We first tested differences in the sizes of the left and right parts of the whole brain and brain morphological regions using a phylogenetic *t*-test when one side was larger than the other. Next, we built PGLS models, treating the index of asymmetry of the whole brain and brain regions as the dependent variable and whole brain size as the independent variable. This approach tested the prediction of the Ringo hypothesis that there were significant associations between the index of brain size asymmetry and the total brain size while controlling for the potential confounding effect of body size. In addition, we analyzed the associations between the index of brain size asymmetry and predation risk. Finally, we examined the differences between the left and right parts of the whole brain and brain regions at intraspecific or inter-specific levels for 99 species. All numeric variables were standardized before analysis to make parameter estimates comparable, and model assumptions were checked and met. Sample sizes varied between models because not all variables were available for all species.

## 3. Results

**Figure 4:**
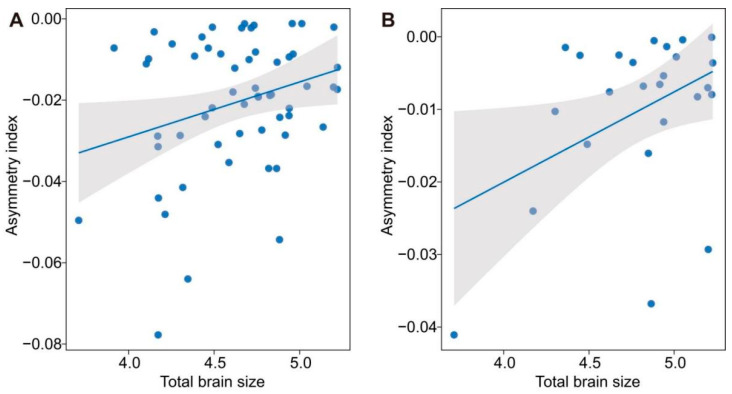
Relationships between asymmetry index of olfactory bulb (**A**) and optic tecta (**B**) and total brain size in species in which the left hemisphere was larger than the right one.

**Figure 5:**
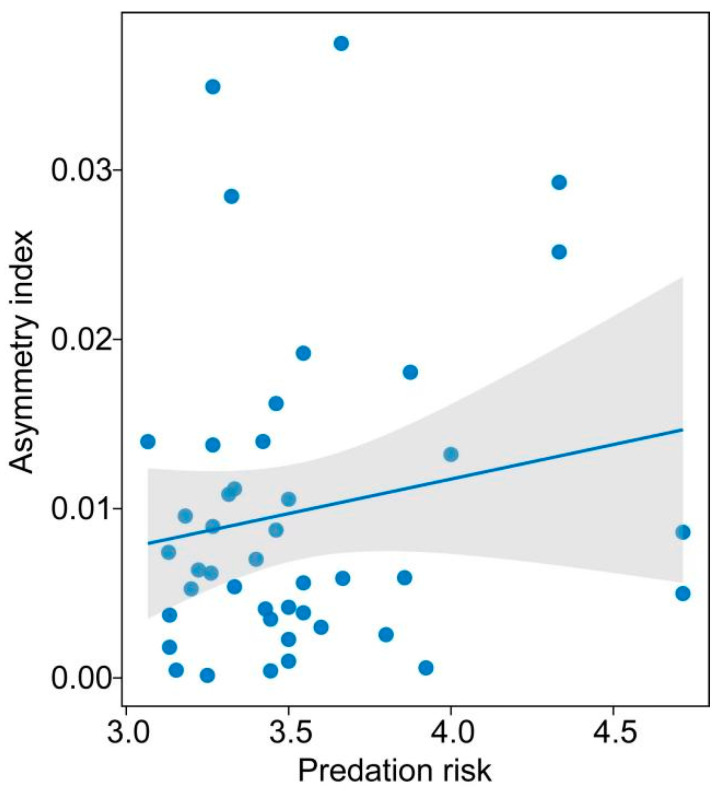
Relationship between the asymmetry index of telencephalon and predation risk in species in which the left hemisphere is smaller than the right one.

We first analyzed differences in the size of the whole brain among 99 species when controlling for the SVL effect and found significant differences in the relative size of the whole brain among species of anurans (ANCOVA: *F*_99,458_ = 4.242, *p* < 0.001). We then analyzed differences between the left and right hemisphere sizes of the whole brain and brain regions in species in which the left hemisphere was larger than the right one. We found a significant difference between the left and right hemispheres of the whole brain and brain regions (Table 1). A PGLS analysis revealed that the index of size asymmetry of the olfactory bulb and optic tecta was positively correlated with total brain size after accounting for the effect of SVL (Table 2; Figure 4). Inconsistent with the prediction of the Ringo hypothesis, the asymmetry index of the total brain size tended to be correlated with the total brain size. Conversely, we observed significant differences between the left and right hemisphere sizes of the whole brain and brain regions in species in which the right hemisphere was larger than the left one (Table 3). Meanwhile, there were no correlations between the index of size asymmetry of the total brain and the three brain regions and the total brain size (Table S3). Additionally, we found no significant correlations between the index of asymmetry of the total brain and the brain regions and predation risk when the left hemisphere was larger than the right one (Table S4). By contrast, there was a positive correlation between the index of asymmetry of telencephalon size and the predation risk in species in which the right hemisphere was larger than the left one (Table 4; Figure 5).

We combined data from all 99 anuran species and analyzed differences between the left and right hemisphere sizes of the whole brain and brain regions. We found non-significant differences between the left and right hemisphere sizes of the whole brain (Table S5). Additionally, the sizes of three main brain regions (i.e., olfactory bulbs, telencephalon, and optic tectum) showed non-significant differences between the left and right hemispheres (Table S5). 

Due to the non-significant left–right differences in the whole brain and brain regions across all 99 species, we decided not to test the relationships between the degree of asymmetry of the whole brain and brain regions and brain size for all species, as well as the relationships between the index of size asymmetry of the whole brain and brain regions and the predation risk. 

## 4. Discussion

We first examined the differences between the left and right hemisphere sizes of the whole brain and brain regions and assessed the associations between the index of asymmetry of the whole brain and brain regions with the total brain size or predation risk across anuran species. We found positive correlations between the index of size asymmetry of the olfactory bulb and optic tecta and the total brain size in species in which the left brain hemisphere was larger than the right one. We also found a positive correlation between the index of asymmetry of the telencephalon size and predation risk when the right hemisphere was larger than the left one. Our study confirmed non-significant differences between the sizes of the left and right sides of the whole brain and three main brain regions among 99 anuran species. 

Most biological systems exhibit varied degrees of asymmetry in the size of morphological organs [74,75,76]. This morphological asymmetry in animals includes both directional and fluctuating asymmetry, which reflects developmental stability under different environmental conditions [6,7,8,77,78,79,80,81]. In humans and many other animals, the two brain hemispheres show significant differences in anatomy and function. Although previous studies on frog brain asymmetry have suggested that sex hormones may influence habenular structural asymmetry [56], no systematic research addresses the relationships between anuran brain asymmetry and environmental factors. In this study, we first observed that anuran species displayed directional asymmetry of the whole brain and brain regions, with one side of the brain being consistently larger than the other, reflecting developmental stability. However, when combining data from all 99 species to analyze the differences between the left and right hemispheres, we found non-significant differences between the sizes of the left and right sides of the whole brain and three main brain regions among anuran species. 

The Ringo hypothesis predicts that larger-brained species exhibit more pronounced laterality compared to smaller-brained species [80]. Interhemispheric conduction delay, which depends on the length of the fiber tracts connecting the two brain hemispheres, is a key factor shaping the evolution of hemispheric asymmetry in mammals [42]. Consequently, longer fiber tracts lead to greater interhemispheric conduction delays, requiring faster reactions to environmental demands and influencing evolutionary pressures controlled by unilateral neural networks. Hence, larger-brained species tend to exhibit more right-lateralized individuals [43,44]. However, inconsistent with the prediction of the Ringo hypothesis [80], the evidence of size asymmetry in the small brains of insects shows that brain size is not an important factor in shaping the evolution of brain size asymmetry [81,82,83]. We found that larger-brained species did not display a larger index of asymmetry in the whole brain compared to smaller-brained species when the left brain hemisphere was larger than the right one. This pattern suggests that the evolution of brain size asymmetry is not influenced by total brain size. However, we found a higher index of size asymmetry of the olfactory bulb and optic tecta in larger-brained species, suggesting that an interhemispheric conduction delay in total brain size might drive the evolution of hemispheric asymmetry of the two brain regions [80]. 

The lateralized specialization of brain size is believed to originate from a combination of developmental, evolutionary, genetic, and environmental factors [47,84,85,86,87,88,89,90,91,92,93,94,95,96,97,98,99,100,101,102]. In particular, the way the preferential use of the left and right eyes affects visual discrimination learning and detour behavior in varied vertebrates, including chicks, fish, anurans, and sheep [85]. Similarly, ultrasonic calls emitted by young mice, which are designed to elicit maternal care, are preferentially processed by the left hemisphere of the mother’s brain [103]. The left hemisphere has also been implicated in generating or perceiving calls in primates and mice, suggesting a conserved role in individual communication [102]. Meanwhile, brain lateralization is associated with learning and memory retrieval, such as the differentiation between short- and long-term memory [104,105,106]. For instance, honeybees favor the right antenna for short-term memory recall, while the left antenna is preferred for long-term memory [106]. Similarly, mice with impaired hippocampal asymmetry exhibit decreased performance in spatial learning and working memory [104]. Moreover, brain lateralization also influences stimuli perception and motor responses [106]. For example, toads are more likely to attack conspecifics on their left side and prey on their right side [107], while poeciliid fish show a consistent rightward turning bias when navigating an opaque barrier but a leftward bias when evading a simulated predator [54]. In our study, 56 species with left-brain lateralization appear to be related to stimuli perception, and future behavioral experiments need to confirm this consistent conclusion. 

There is evidence that the left and right hemispheres of the whole brain and brain regions generally develop with a high degree of symmetry at both the anatomical and functional levels in most animals [1,2,3]. However, subtle structural differences between the two sides can influence the processing of specific cognitive tasks [92]. Hemispheric specialization for particular cognitive functions likely reflects variations in the neural circuits of each hemisphere [3]. For example, light-induced visual lateralization has been shown to enhance chicks’ performance in dual tasks involving predator monitoring [108]. Similarly, the bias in front paw use is inversely correlated with the side of the face displaying supernumerary whiskers in mice, a phenomenon that has been suggested to result from competition for cortical space between motor and somatosensory areas [1]. Our findings for the directional size asymmetry of the whole brain and brain regions across anuran species suggest that specific cognitive tasks are closely tied to the neural circuits of each brain hemisphere. Similar findings have been reported for asymmetry in amphibia [109,110,111].

Although interhemispheric conduction delay is an important factor in shaping the evolution of brain hemispheric asymmetry, environmental factors must also be considered in this context. Previous studies have demonstrated that sociality plays a crucial role in both the evolution of brain size [112] and population-level brain hemispheric asymmetry [112,113,114,115]. Consequently, inter-individual interactions produce evolutionarily stable strategies of lateralization, depending on environmental conditions [116], which is supported by empirical evidence from insects [117] and fish [118]. For instance, social honeybees display brain hemispheric asymmetry on both behavioral and electrophysiological levels, while non-social bees do not [117]. For anuran species, sociality linked to anti-predator risk cannot play an important role in shaping the evolution of brain hemispheric asymmetry in breeding season [20,119,120]. In addition, the digit ratio, which is an indicator of brain laterality, is associated with behaviors in the gecko *Ptyodactylus guttatus* [8]. Indeed, we found that there were non-significant differences between the left and right hemispheres of the whole brain and brain regions across 99 species. However, we found that the index of asymmetry of telencephalon size was correlated positively with predation risk in species in which the right hemisphere was larger than the left one, suggesting that an increasing predation risk linked to sociality possibly promoted the enlarged right telencephalon. 

## 5. Conclusions

This study indicated that anurans exhibit significant differences between the left and right hemispheres of the whole brain and brain regions across various anuran species when one side of the brain is larger than the other. Meanwhile, the index of size asymmetry of the olfactory bulb and optic tecta is positively correlated with total brain size in species in which the left brain hemisphere is larger than the right one, suggesting that interhemispheric conduction delays shaped the evolution of the hemispheric asymmetry of the olfactory bulb and optic tecta. Moreover, the positive and significant correlation between the index of asymmetry of the telencephalon size and predation risk in species in which the right brain hemisphere was larger than the left one suggests that the increased predation risk promoted the increased right telencephalon. 

## Figures and Tables

**Table 1:** Differences in sizes of the left and right hemispheres of the whole brain and brain regions (mean ± SD) in anuran species in which the left hemisphere was larger than the right one using phylogenetic paired *t*-test.

Brain Regions	Left Hemisphere (mm^3^)	Right Hemisphere (mm^3^)	*t*	*df*	*p*
Olfactory bulb	2.745 ± 0.387	2.692 ± 0.393	10.636	54	<0.001
Telencephalon	3.678 ± 0.338	3.641 ± 0.348	8.802	53	<0.001
Optic tecta	3.500 ± 0.395	3.467 ± 0.405	2.202	22	0.038
Total brain	4.351 ± 0.362	4.315 ± 0.368	10.280	56	<0.001

**Table 2:** PGLS models assessing the relationships between asymmetry index of the total brain and three main brain regions among anuran species in which the left hemisphere is larger than the right one. Snout–vent length (SVL) was added as a covariate. Phylogenetic scaling parameters (superscripts following λ denote *p*-values of likelihood ratio tests against models with λ = 0 and 1, respectively).

Dependent Variable	Brain Regions	Independent Variable	*λ*	*R* ^2^	*β*	*t*	*p*
Asymmetry index	Olfactory bulb	Total brain	<0.001^(<0.001, 0.915)^	0.090	0.038	2.349	0.022
		SVL			−0.054	−1.636	0.108
	Telencephalon	Total size	<0.0001^(<0.001, 0.348)^	0.128	0.019	1.801	0.077
		SVL			−0.017	−0.830	0.410
	Optic tecta	Total brain	0.460^(<0.001, 0.952)^	0.140	0.035	2.378	0.027
		SVL			−0.059	−1.975	0.061
	Total brain	Total size	0.036^(<0.001, 0.396)^	0.105	0.013	1.909	0.061
		SVL			−0.013	−0.944	0.349

**Table 3:** Results of phylogenetic paired t-tests for the sizes of the left and right hemispheres of the whole brain and brain regions (mean ± SD) in anuran species in which the left hemisphere was smaller than the right one.

Brain Regions	Left Hemisphere (mm^3^)	Right Hemisphere (mm^3^)	*t*	*df*	*p*
Olfactory bulb	2.684 ± 0.416	2.739 ± 0.405	−5.556	39	<0.001
Telencephalon	3.759 ± 0.294	3.796 ± 0.292	−2.517	40	0.016
Optic tecta	3.323 ± 0.343	3.373 ± 0.339	−6.322	71	<0.001
Total brain	4.365 ± 0.341	4.397 ± 0.341	−4.077	37	<0.001

**Table 4:** PGLS models assessing the relationship between asymmetry index of the whole brain and three brain regions and predation risk in species in which the left hemisphere is smaller than the right one. Snout–vent length (SVL) was added as a covariate. Phylogenetic scaling parameters (superscripts following λ denote *p*-values of likelihood ratio tests against models with λ = 0 and 1, respectively).

Dependent Variable	Brain Regions	Independent Variable	*λ*	*R* ^2^	*β*	*t*	*p*
Asymmetry index	Olfactory bulb	Predation risk	<0.001^(<0.001, 0.332)^	0.070	−0.022	−1.785	0.082
		SVL			−0.049	−2.171	0.036
	Telencephalon	Predation risk	1^(0.032, <0.001)^	0.077	0.008	2.289	0.027
		SVL			0.016	1.600	0.118
	Optic tecta	Predation risk	0.060^(<0.001, 0.434)^	0.017	0.002	0.335	0.739
		SVL			−0.014	−1.297	0.199
	Total brain	Predation risk	0.262^(0.034, 0.676)^	−0.015	0.003	1.080	0.287
		SVL			0.006	1.019	0.315

## Data Availability

The data presented in this study are available on request from the corresponding author.

## Supplementary Materials

The following supporting information can be downloaded at: https://www.mdpi.com/article/10.3390/biology14010038/s1, Table S1: Species, line length (m), line wide (m), line square (m^2^) and snake species (individuals) in anuran species. Table S2: Species, body size, size of left and right brain hemispheres of total brain and three main brain regions and predation risk in anuran species; Table S3: the relationships between asymmetry index of total brain and three brain regions and the total brain size in species where the left hemisphere is smaller than the right one; Table S4: the relationships between the asymmetry index of different brain regions and predation risk in species where the left hemisphere is larger than the right hemisphere; Table S5: Difference in sizes of the left and right hemisphere sizes in total brain and three brain regions among 99 anuran species.
