# Supplementary material for: Exploring Brain Size Asymmetry and Its Relationship with Predation Risk Among Chinese Anurans
Source: Biology (Basel). 2025 Jan 7;14(1):38. doi: 10.3390/biology14010038 (PMC11762737; doi:10.3390/biology14010038)
Supplement: Supplementary file 1 [file biology-14-00038-s001.zip › biology-3261752-supplementary.pdf]

Table S1 Species, line length (m), line wide (m), line square (m^2) and snake species (individuals) in anuran species.

Species	Line length (m)	Line wide (m)	Line square (m^2)	Snake species (individuals)
Amolops_chunganensis	1000	5	5000	*Cyclophiops major* (1)*, Trimeresurus stejnegeri* (1)
Amolops_granulosus	1100	5	5500	*Cyclophiops major* (1)*, Trimeresurus stejnegeri* (2)
Amolops_lifanensis	900	5	4500	*Gloydius strauchii* (2)
Amolops_loloensis	1200	5	6000	*Trimeresurus stejnegeri* (1)
Amolops_mantzorum	1800	5	9000	*Trimeresurus stejnegeri* (1)*, Rhabdophis tigrinus* (1)
Amolops_ricketti	1100	5	5500	*Protobothrops mucrosquamatus* (2)
Amolops_sinensis	1400	5	7000	*Lycodon rufozonatus* (2)
Amolops_torrentis	3000	5	15000	*Trimeresurus stejnegeri* (3)
Amolops_yunkaiensis	1500	5	7500	*Lycodon ruhstrati* (2)
Aquixalus_palpebralis	2200	5	11000	*Ovophis tonkinensis* (1)*, Trimeresurus stejnegeri* (1)
Babina_daunchina	800	5	4000	*Trimeresurus stejnegeri* (1)*, Dinodon rufozonatum* (3)
Bombina_maxima	700	5	3500	*Dinodon rufozonatum* (1)*, Cyclophiops major* (2)
Brachytarsophrys_chuannanensis	2600	5	13000	*Protobothrops jerdonii* (2)
Bufo_andrewsi	3000	5	15000	*Protobothrops mucrosquamatus* (1)*, Dinodon rufozonatum* (1)
Bufo_gargarizans	1500	5	7500	*Dinodon rufozonatum* (1)

Bufo_melanostictus	1100	5	5500	*Gloydius cognatus* (1)
Bufo_minshanicus	1500	5	7500	*Rhabdophis tigrinus(2)*
Bufo_raddei	1500	5	7500	*Gloydius strauchii* (1)
Bufo_tibetanus	500	5	2500	*Dinodon rufozonatum* (2)
Calluella_yunnanensis	1000	5	5000	*Lycodon fasciatus* (2), *Trimeresurus stejnegeri* (1)
Chirixalus_doriae	600	5	3000	*Lycodon ruhstrati* (2)
Chirixalus_vittatus	2500	5	12500	*Trimeresurus stejnegeri* (1)
Feirana_quadranus	1000	5	5000	*Elaphe taeniura* (1), *Protobothrops mucrosquamatus* (1)
Fejervarya_multistriata	1500	5	7500	*Trimeresurus stejnegeri* (1), *Dinodon rufozonatum* (3)
Hyla_annectans_jingdongensis	800	5	4000	*Gloydius brevicaudus* (1), *Trimeresurus stejnegeri* (2)
Hyla_annectans_wulingensis	1000	5	5000	*Trimeresurus stejnegeri* (4)
Hyla_sanchiangensis	1000	5	5000	*Trimeresurus stejnegeri* (2)
Hyla_tsinlingensis	1100	5	5500	*Elaphe taeniura* (1)
Hylarana_guentheri	1600	5	8000	*Rhabdophis tigrinus*(3), *Enhydris plumbea* (1)
Hylarana_nigrotympanica	2000	5	10000	*Lycodon fasciatus, Enhydris plumbea(2)*
Kaloula_pulchra	400	5	2000	*Dinodon rufozonatum* (2)
Kaloula_rugifera	500	5	2500	*Trimeresurus stejnegeri* (2)
Kaloula_verrucosa	600	5	3000	*Dinodon rufozonatum* (2)
Leptobrachella_bijie	1000	5	5000	*Dinodon rufozonatum* (2), *Trimeresurus stejnegeri* (1)
Leptobrachium_boringii	900	5	4500	*Protobothrops jerdonii* (2)

Leptobrachella_feii	2000	5	10000	*Lycodon fasciatus* (3), *Enhydris plumbea* (2)
Leptolalax_oshanensis	2000	5	10000	*Lycodon fasciatus* (1), *Enhydris plumbea* (2), *Bungarus fasciatus* (1), *Trimeresurus stejnegeri* (3)
Leptolalax_ventripunctatus	1500	5	7500	*Trimeresurus stejnegeri* (2)
Limnonectes_fujianensis	1800	5	9000	*Elaphe taeniura* (1), *Protobothrops mucrosquamatus* (2)
Lithobates_catesbeianus	2300	5	11500	*Dinodon rufozonatum* (2),*Trimeresurus stejnegeri* (2)
Megophrys_daweimontis	500	5	2500	*Rhabdophis subminiatus* (2)
Megophrys_jingdongensis	600	5	3000	*Ovophis tonkinensis* (4), *Trimeresurus stejnegeri* (1)
Megophrys_kuatunensis	1500	5	7500	*Dinodon rufozonatum* (1), *Trimeresurus stejnegeri* (1)
Megophrys_major	600	5	3000	*Zaocys dhumnades* (3), *Cyclophiops major* (1)
Megophrys_minor	1200	5	6000	*Achalinus spinalis* (1), *Cyclophiops major* (1)
Megophrys_omeimontis	1000	5	5000	*Enhydris plumbea* (2), *Bungarus fasciatus* (2), *Lycodon fasciatus* (1)
Megophrys_parva	1500	5	7500	*Trimeresurus stejnegeri* (3), *Dinodon rufozonatum* (4)
Megophrys_shapingensis	400	5	2000	*Bungarus fasciatus* (2), *Lycodon fasciatus* (1)
Megophrys_spinata	450	5	2250	*Lycodon rufozonatus* (2), *Dinodon rufozonatum* (1)
Microhyla_berdmorei	600	5	3000	*Dinodon rufozonatum* (4), *Pareas chinensis* (1), *Elaphe taeniura* (1)
Microhyla_butleri	500	5	2500	*Lycodon rufozonatus* (1), *Dinodon rufozonatum* (2)
Microhyla_heymonsi	500	5	2500	*Elaphe taeniura* (2), *Dinodon rufozonatum* (1)

Microhyla_mixtura	700	5	3500	*Dinodon rufozonatum* (1),*Pareas chinensis* (3)
Microhyla_ornata	500	5	2500	*Gloydius strauchii* (1), *Protobothrops jerdonii* (1)
Microhyla_pulchra	1200	5	6000	*Dinodon rufozonatum* (3)
Nanorana_pleskei	1100	5	5500	*Trimeresurus stejnegeri* (2), *Dinodon rufozonatum* (1)
Nidirana_yeae	900	5	4500	*Amphiesma sauteri* (2), *Trimeresurus stejnegeri* (1)
Occidozyga_martensii	1000	5	5000	*Amphiesma sauteri* (2), *Trimeresurus stejnegeri* (2)
Odorrana_grahami	1900	5	9500	*Trimeresurus stejnegeri* (1), *Achalinus spinalis* (1), *Ramphotyphlops braminus* (1)
Odorrana_graminea	1200	5	6000	*Lycodon ruhstrati* (2), *Bungarus fasciatus* (1)
Odorrana_hainanensis	1300	5	6500	*Lycodon ruhstrati* (2), *Bungarus fasciatus* (1)
Odorrana_hejiangensis	3000	5	15000	*Trimeresurus stejnegeri* (1)
Odorrana_huanggangensis	1100	5	5500	*Trimeresurus stejnegeri* (2), *Dinodon rufozonatum* (1)
Odorrana_kuangwuensis	1600	5	8000	*Trimeresurus stejnegeri* (2), *Dinodon rufozonatum* (2), *Protobothrops jerdonii* (3)
Odorrana_kweichowensis	1300	5	6500	*Deinagkistrodon acutus* (2), *Lycodon rufozonatus* (1)
Odorrana_lungshengensis	1100	5	5500	*Trimeresurus stejnegeri* (2), *Dinodon rufozonatum* (1)
Odorrana_margaretae	2300	5	11500	*Deinagkistrodon acutus* (1), *Lycodon rufozonatus* (1), *Bungarus fasciatus* (1)
Odorrana_nanjiangensis	1500	5	7500	*Protobothrops jerdonii* (1), *Trimeresurus stejnegeri* (2)
Odorrana_nasuta	1200	5	6000	*Lycodon ruhstrati* (2), *Bungarus fasciatus* (1)

Odorrana_schmackeri	1300	5	6500	*Elaphe taeniura* (2), *Dinodon rufozonatum* (1)
Odorrana_tiannanensis	1800	5	9000	*Dinodon rufozonatum* (3), *Trimeresurus stejnegeri* (3)
Occidozyga_lima	1000	5	5000	*Dinodon rufozonatum* (3), *Trimeresurus stejnegeri* (2)
Ophryophryne_microstoma	1600	5	8000	*Cyclophiops major* (1), *Trimeresurus stejnegeri* (1), *Elaphe taeniura* (2)
Oreolalax_rugosus	1400	5	7000	*Trimeresurus stejnegeri* (1), *Elaphe taeniura* (2)
Paa_boulengeri	1100	5	5500	*Dinodon rufozonatum* (1), *Cyclophiops major* (1)
Paa_robertingeri	1900	5	9500	*Trimeresurus stejnegeri* (1), *Achalinus spinalis* (2), *Ramphotyphlops braminus* (1)
Paa_sichuanensis	700	5	3500	*Dinodon rufozonatum* (2), *Elaphe taeniura* (1)
Paa_yunnanensis	1800	5	9000	*Dinodon rufozonatum* (1)
Pelophylax_hubeiensis	3000	5	15000	*Trimeresurus stejnegeri* (1), *Achalinus spinalis* (1)
Pelophylax_nigromaculatus	1500	5	7500	*Trimeresurus stejnegeri* (1), *Dinodon rufozonatum* (3), *Ptyas mucosus* (4)
Pelophylax_pleuraden	1400	5	7000	*Rhabdophis subminiatus* (3), *Ptyas mucosus* (2)
Philautus_gracilipes	1300	5	6500	*Trimeresurus stejnegeri* (2), *Ptyas mucosus* (3)
Polypedates_impresus	1300	5	6500	*Trimeresurus stejnegeri* (3), *Rhabdophis subminiatus* (3)
Polypedates_megacephalus	1000	5	5000	*Elaphe taeniur* (2), *Zaocys dhumnades* (2)
Polypedates_mutus	1000	5	5000	*Elaphe taeniur* (1), *Zaocys dhumnades* (3), *Dinodon rufozonatum* (2)

Pseudorana_weiningensis	700	5	3500	*Cyclophiops major* (1)*, Trimeresurus stejnegeri* (2), *Pseudoxenodon macrops* (3)
Rana_chaochiaoensis	1200	5	6000	*Achalinus spinalis*(2)*, Hebius sauteri* (1)*, Protobothrops jerdonii* (1)
Rana_chensinensis	1400	5	7000	*Elaphe taeniura* (1)*, Zaocys dhumnades* (1)
Rana_hanluica	900	5	4500	*Trimeresurus stejnegeri* (2)
Rana_kukunoris	2000	5	10000	*Gloydius cognatus* (2)
Rana_omeimontis	2500	5	12500	*Hebius craspedogaster* (1), *Ptyas major* (2)
Rhacophorus_chenfui	600	5	3000	*Protobothrops jerdonii* (3)*, Pseudoxenodon macrops* (1)
Rhacophorus_dennysi	4600	5	23000	*Zaocys dhumnades* (2)*, Trimeresurus stejnegeri* (1)
Rhacophorus_dugritei	1300	5	6500	*Achalinus meiguensis* (1), *Cyclophiops major* (1)
Rhacophorus_feae	3200	5	16000	*Dinodon rufozonatum* (2)
Rhacophorus_omeimontis	2400	5	12000	*Protobothrops jerdonii* (2), *Cyclophiops major* (1)
Scutiger_chintingensis	1100	5	5500	*Hebius craspedogaster* (1), *Ptyas major* (2)
Xenophrys_leishanensis	800	5	4000	*Trimeresurus stejnegeri* (2), *Cyclophiops major* (3)
Xenophrys_wushanensis	1000	5	5000	*Dinodon rufozonatum* (1), *Trimeresurus stejnegeri* (1)

Table S2 Species, sampling size (n), body size (mm), size of left and right brain hemispheres of total brain and three main brain regions (mm3) and predation pressure (numbers/km2) in anuran species.

Species	Log (SVL)	n	Olfactory bulb		Telencephalon		Optic tecta		Total brain		Predation risk
			Left Mean (SD)	Right Mean (SD)	Left Mean (SD)	Right Mean (SD)	Left Mean (SD)	Right Mean (SD)	Left Mean (SD)	Right Mean (SD)	
Amolops_chunganensis	4.591	6	0.574 (0.470)	0.481 (0.400)	4.698 (2.849)	4.087 (2.295)	2.668 (1.491)	2.780 (1.646)	23.806 (18.045)	20.788 (13.281)	0.400
Amolops_granulosus	4.513	1	0.833	0.720	3.269	2.678	1.950	2.460	16.233	14.582	0.546
Amolops_lifanensis	4.723	1	0.758	0.647	8.570	7.555	4.188	4.196	40.099	36.393	0.167
Amolops_loloensis	4.818	4	1.022 (1.208)	0.869 (0.614)	8.850 (1.924)	9.381 (1.916)	4.672 (1.971)	4.465 (0.700)	43.021 (11.916)	43.595 (13.318)	0.222
Amolops_mantzorum	4.797	8	0.698 (0.517)	0.580 (0.414)	10.522 (2.039)	10.147 (1.223)	4.594 (0.824)	4.347 (0.423)	43.907 (7.799)	38.447 (5.413)	0.364
Amolops_ricketti	4.721	7	0.684 (0.224)	0.605 (0.294)	5.736 (1.078)	5.730 (1.616)	3.482 (1.402)	3.563 (1.807)	29.486 (11.153)	27.547 (12.526)	0.286
Amolops_sinensis	4.721	6	0.450	0.493	5.042	5.274	2.876	2.931	23.120	23.467	0.200

			(0.395)	(0.383)	(1.061)	(1.162)	(1.008)	(1.155)	(5.201)	(4.106)	
Amolops_torrentis	4.767	3	0.698<br>(0.659)	0.711<br>(0.168)	5.451<br>(2.420)	5.335<br>(1.923)	4.330<br>(2.219)	3.790<br>(2.547)	34.4825<br>(34.287)	36.085<br>(15.462)	0.267
Amolops_yunkaiensis	4.767	2	0.461	0.457	4.953	5.375	3.454	3.520	28.150	25.943	0.182
Aquixalus_palpebralis	4.463	5	0.143<br>(0.186)	0.105<br>(0.149)	3.157<br>(1.268)	2.456<br>(1.043)	1.569<br>(0.931)	1.583<br>(0.915)	12.305<br>(11.553)	9.901<br>(4.023)	1.000
Babina_daunchina	4.684	4	0.299<br>(0.133)	0.285<br>(0.097)	6.096<br>(1.187)	6.420<br>(2.525)	3.135<br>(1.353)	3.235<br>(1.847)	27.049<br>(8.344)	28.116<br>(33.368)	0.857
Bombina_maxima	4.742	8	0.944<br>(0.157)	0.744<br>(0.302)	6.646<br>(1.099)	5.422<br>(1.507)	1.260<br>(0.088)	1.295<br>(0.364)	20.757<br>(6.797)	17.827<br>(3.435)	0.126
Brachytarsophrys_chuannanensis	5.008	1	0.584	0.417	5.754	7.388	3.230	3.216	34.790	41.418	0.324
Bufo_andrewsi	4.925	8	1.724<br>(1.452)	1.768<br>(1.578)	14.074<br>(10.253)	14.136<br>(8.863)	3.741<br>(2.750)	3.834<br>(2.648)	52.459<br>(48.460)	53.937<br>(41.371)	0.154
Bufo_gargarizans	5.046	8	3.220<br>(1.406)	3.387<br>(1.266)	21.812<br>(5.998)	22.638<br>(7.727)	7.029<br>(1.751)	6.809<br>(2.549)	87.486<br>(28.001)	80.895<br>(56.394)	0.133
Bufo_melanostictus	4.758	8	1.519<br>(0.542)	1.506<br>(0.456)	13.376<br>(6.771)	12.576<br>(5.755)	4.007<br>(2.326)	3.916<br>(1.451)	51.449<br>(17.755)	51.463<br>(15.620)	0.133
Bufo_minshanicus	4.827	8	1.453<br>(1.858)	1.345<br>(1.746)	12.208<br>(13.357)	11.376<br>(11.249)	2.392<br>(1.606)	2.683<br>(1.005)	37.396<br>(37.366)	36.382<br>(33.451)	0.182
Bufo_raddei	4.770	3	0.594	0.738	5.240	5.659	1.477	1.602	17.254	16.443	0.267

			(0.206)	(0.258)	(0.915)	(0.614)	(0.725)	(0.442)	(2.952)	(3.904)	
Bufo_tibetanus	4.768	5	1.594<br>(0.446)	1.391<br>(0.319)	10.989<br>(1.057)	10.276<br>(3.129)	2.486<br>(0.563)	2.575<br>(0.395)	35.049<br>(5.286)	33.212<br>(28.192)	0.133
Calluella_yunnanensis	4.512	4	0.159<br>(0.191)	0.180<br>(0.272)	2.953<br>(1.193)	2.703<br>(0.797)	0.576<br>(0.091)	0.623<br>(0.202)	9.753<br>(9.542)	9.002<br>(3.243)	0.800
Chirixalus_doriae	4.389	5	0.248<br>(0.209)	0.251<br>(0.180)	2.278<br>(0.260)	2.108<br>(0.220)	1.1699<br>(0.175)	1.259<br>(0.380)	8.691<br>(2.192)	8.962<br>(2.273)	0.600
Chirixalus_vittatus	4.504	2	0.097<br>(0.061)	0.069<br>(0.028)	1.886<br>(0.122)	1.681<br>(0.207)	0.820<br>(0.178)	0.863<br>(0.088)	7.811<br>(1.177)	7.067<br>(2.048)	0.667
Feirana_quadranus	4.915	9	1.915<br>(1.298)	1.750<br>(0.982)	20.179<br>(13.824)	18.957<br>(9.721)	8.215<br>(4.760)	7.649<br>(3.864)	86.378<br>(47.298)	79.765<br>(38.323)	0.080
Fejervarya_multistriata	4.598	8	0.743<br>(0.368)	0.660<br>(0.302)	3.805<br>(1.428)	3.457<br>(1.343)	2.892<br>(0.694)	3.020<br>(0.519)	20.737<br>(6.606)	19.993<br>(15.31)	0.400
Hyla_annectans_jingdongensis	4.525	4	0.279<br>(0.331)	0.307<br>(0.161)	2.922<br>(1.996)	2.814<br>(1.083)	1.204<br>(0.633)	1.192<br>(0.122)	12.036<br>(2.088)	10.966<br>(11.247)	0.533
Hyla_annectans_wulingensis	4.553	8	0.305<br>(0.064)	0.318<br>(0.114)	3.923<br>(1.088)	3.872<br>(1.260)	1.351<br>(0.434)	1.414<br>(0.715)	12.069<br>(3.322)	11.669<br>(4.076)	0.750
Hyla_sanchiangensis	4.530	4	0.182<br>(0.174)	0.147<br>(0.174)	3.439<br>(0.281)	3.291<br>(0.24)	1.160<br>(0.335)	1.389<br>(0.174)	9.936<br>(2.128)	10.794<br>(1.297)	0.800
Hyla_tsinlingensis	4.532	5	0.376	0.326	5.206	4.920	1.788	1.754	14.961	13.059	0.400

			(0.238)	(0.164)	(2.026)	(0.597)	(0.939)	(0.657)	(5.828)	(3.569)	
Hylarana_guentheri	4.699	8	1.562<br>(1.165)	1.331<br>(1.115)	8.639<br>(3.044)	7.982<br>(3.044)	5.416<br>(1.683)	5.643<br>(1.762)	45.054<br>(13.598)	41.831<br>(13.111)	0.182
Hylarana_nigrotympanica	4.643	2	0.870<br>(1.063)	0.813<br>(1.020)	4.492<br>(0.409)	4.579<br>(1.402)	2.76<br>(0.706)	3.096<br>(0.835)	25.715<br>(8.486)	24.887<br>(6.890)	0.500
Kaloula_pulchra	4.886	2	2.402<br>(1.016)	1.813<br>(0.607)	11.155<br>(4.935)	10.852<br>(7.538)	3.436<br>(1.051)	2.562<br>(2.322)	36.791<br>(4.702)	36.510<br>(9.442)	0.300
Kaloula_rugifera	4.462	2	0.497<br>(0.056)	0.417<br>(0.265)	3.692<br>(1.048)	2.799<br>(0.618)	0.707<br>(0.088)	0.662<br>(0.008)	11.329<br>(13.782)	8.683<br>(0.059)	1.000
Kaloula_verrucosa	4.632	4	0.626<br>(0.295)	0.618<br>(0.21)	4.392<br>(2.879)	4.313<br>(1.057)	1.322<br>(0.950)	1.189<br>(0.525)	16.039<br>(6.417)	14.852<br>(4.437)	0.800
Leptobrachella_bijie	4.452	4	0.097<br>(0.055)	0.106<br>(0.090)	2.014<br>(0.898)	2.107<br>(0.880)	0.814<br>(0.445)	0.934<br>(0.406)	8.732<br>(10.313)	8.664<br>(2.040)	0.667
Leptobrachium_boringii	4.645	2	0.393<br>(0.006)	0.431<br>(0.332)	7.626<br>(4.035)	7.833<br>(2.105)	2.558<br>(0.569)	2.744<br>(0.296)	30.357<br>(9.496)	29.654<br>(9.520)	0.600
Leptobrachella_feii	4.228	4	0.102<br>(0.042)	0.127<br>(0.085)	1.548<br>(0.457)	1.588<br>(0.427)	0.588<br>(0.187)	0.870<br>(0.381)	6.404<br>(2.990)	7.271<br>(3.336)	0.444
Leptolalax_oshanensis	4.429	4	0.1009<br>(0.067)	0.114<br>(0.120)	2.049<br>(0.487)	2.116<br>(0.376)	0.817<br>(0.335)	0.967<br>(0.547)	7.879<br>(1.501)	7.851<br>(0.758)	0.500
Leptolalax_ventripunctatus	4.413	3	0.131	0.106	1.782	1.572	0.851	0.878	7.321	7.621	0.700

			(0.124)	(0.055)	(0.595)	(0.427)	(0.188)	(0.302)	(1.205)	(1.005)	
Limnonectes_fujianensis	4.681	4	0.365 (0.525)	0.694 (1.197)	4.413 (2.618)	5.946 (3.894)	3.073 (1.496)	3.397 (2.110)	30.293 (23.266)	34.735 (23.985)	0.267
Lithobates_catesbeianus	5.073	8	3.969 (3.710)	4.057 (4.326)	18.087 (13.943)	19.071 (17.246)	12.617 (5.056)	13.194 (6.853)	118.261 (101.018)	121.803 (110.754)	0.333
Megophrys_daweimontis	4.441	1	0.202	0.265	4.771	4.333	1.396	1.441	16.287	15.657	0.348
Megophrys_jingdongensis	4.474	1	0.142	0.267	3.103	2.853	1.143	1.447	9.360	10.356	0.800
Megophrys_kuatunensis	4.465	4	0.201 (0.070)	0.191 (0.056)	2.008 (0.806)	1.904 (0.615)	0.763 (0.195)	0.824 (0.274)	6.905 (1.873)	6.185 (6.341)	1.667
Megophrys_major	4.708	1	0.708	0.854	8.811	9.993	2.798	4.383	35.188	42.010	0.267
Megophrys_minor	4.619	4	0.263 (0.197)	0.253 (0.190)	3.895 (1.197)	4.979 (1.591)	1.369 (0.577)	1.915 (0.436)	12.529 (1.476)	16.709 (2.522)	1.333
Megophrys_omeimontis	4.781	2	0.534 (0.048)	0.626 (0.336)	7.556 (0.427)	8.353 (1.489)	2.819 (0.497)	3.298 (0.504)	30.230 (1.051)	33.137 (0.016)	0.333
Megophrys_parva	4.583	1	0.293	0.278	3.375	3.759	1.564	1.825	11.302	13.011	1.000
Megophrys_shapingensis	4.861	1	1.170	1.365	4.899	6.778	1.896	2.274	22.316	27.738	0.662
Megophrys_spinata	4.711	4	0.523 (0.301)	0.485 (0.237)	4.254 (1.292)	4.176 (1.800)	1.533 (0.340)	1.451 (0.332)	21.321 (6.830)	20.429 (4.597)	0.933
Microhyla_berdmorei	4.408	1	0.247	0.253	1.946	1.659	0.787	0.911	6.853	6.797	1.500
Microhyla_butleri	4.339	2	0.115	0.115	0.812	0.964	0.346	0.479	4.386	4.073	1.333

			(0.133)	(0.126)	(1.017)	(1.059)	(0.421)	(0.538)	(7.037)	(4.224)	
Microhyla_heymonsi	4.351	3	0.127<br>(0.054)	0.123<br>(0.079)	1.096<br>(0.888)	1.042<br>(0.424)	0.384<br>(0.221)	0.417<br>(0.066)	4.302<br>(3.527)	3.920<br>(3.334)	2.000
Microhyla_mixtura	4.361	3	0.092<br>(0.020)	0.074<br>(0.014)	0.729<br>(0.181)	0.514<br>(0.113)	0.287<br>(0.054)	0.228<br>(0.082)	2.934<br>(3.126)	2.177<br>(2.481)	1.200
Microhyla_ornata	4.329	5	0.156<br>(0.307)	0.173<br>(0.262)	1.406<br>(0.893)	1.458<br>(0.558)	0.473<br>(0.306)	0.501<br>(0.311)	5.158<br>(1.289)	5.202<br>(1.389)	1.714
Microhyla_pulchra	4.521	4	0.301<br>(0.466)	0.252<br>(0.372)	2.360<br>(3.192)	2.027<br>(2.320)	0.598<br>(0.660)	0.514<br>(0.512)	8.183<br>(8.220)	6.680<br>(7.496)	1.456
Nanorana_pleskei	4.503	8	0.407<br>(0.238)	0.396<br>(0.181)	4.158<br>(1.769)	4.248<br>(1.718)	1.011<br>(0.704)	1.231<br>(0.624)	13.419<br>(11.967)	13.440<br>(12.065)	0.800
Nidirana_yeae	4.716	4	0.778<br>(0.126)	0.852<br>(0.157)	5.209<br>(1.468)	5.384<br>(1.764)	3.414<br>(1.210)	3.475<br>(1.051)	27.666<br>(6.132)	29.310<br>(7.633)	0.546
Occidozyga_martensii	4.362	8	0.168<br>(0.063)	0.145<br>(0.042)	1.547<br>(0.382)	1.545<br>(0.525)	0.966<br>(0.235)	0.997<br>(0.235)	7.562<br>(1.673)	7.232<br>(1.826)	0.667
Odorrana_grahami	4.924	8	1.809<br>(1.493)	1.486<br>(1.223)	15.560<br>(4.984)	14.106<br>(5.555)	7.279<br>(2.093)	6.765<br>(2.676)	72.463<br>(33.678)	64.596<br>(30.991)	0.800
Odorrana_graminea	4.850	8	1.153<br>(1.033)	1.0267<br>(0.856)	10.292<br>(9.010)	11.383<br>(11.657)	5.267<br>(4.301)	6.983<br>(5.939)	51.742<br>(46.769)	58.524<br>(63.436)	0.316
Odorrana_hainanensis	4.722	4	1.062	0.879	5.581	5.629	3.872	4.671	30.100	29.965	0.500

			(0.821)	(0.267)	(3.001)	(2.553)	(1.507)	(1.927)	(12.103)	(11.761)	
Odorrana_hejiangensis	4.884	7	1.204<br>(0.918)	1.311<br>(1.017)	13.148<br>(7.764)	13.100<br>(8.239)	5.966<br>(3.678)	6.223<br>(3.630)	57.246<br>(30.984)	54.313<br>(29.389)	0.462
Odorrana_huanggangensis	4.639	4	0.536<br>(0.433)	0.532<br>(0.474)	5.073<br>(1.693)	5.719<br>(2.314)	2.691<br>(0.670)	3.257<br>(0.811)	23.497<br>(7.286)	24.107<br>(7.237)	0.067
Odorrana_kuangwuensis	4.751	1	0.107	0.147	4.612	4.836	1.874	2.331	18.442	17.516	0.546
Odorrana_kweichowensis	4.652	4	0.629<br>(0.462)	0.660<br>(0.301)	4.906<br>(1.142)	5.728<br>(1.370)	4.308<br>(0.651)	4.672<br>(0.929)	32.740<br>(33.611)	31.354<br>(9.072)	0.875
Odorrana_lungshengensis	4.822	5	0.803<br>(0.773)	0.822<br>(0.675)	8.915<br>(4.448)	10.343<br>(4.750)	4.995<br>(0.814)	5.231<br>(1.708)	40.415<br>(8.607)	39.307<br>(7.873)	0.462
Odorrana_margaretae	4.914	8	2.132<br>(0.972)	1.877<br>(0.594)	17.949<br>(7.614)	16.608<br>(7.124)	7.901<br>(2.365)	7.420<br>(1.716)	85.176<br>(39.749)	72.435<br>(33.898)	0.546
Odorrana_nanjiangensis	4.760	4	0.927<br>(0.179)	0.982<br>(0.221)	7.874<br>(1.395)	8.325<br>(1.113)	4.696<br>(1.487)	5.121<br>(0.638)	38.177<br>(9.471)	40.022<br>(8.805)	0.261
Odorrana_nasuta	4.850	6	1.121<br>(0.534)	1.137<br>(0.524)	10.295<br>(8.422)	10.265<br>(6.957)	6.634<br>(4.196)	9.145<br>(11.485)	58.22<br>(41.035)	54.031<br>(49.698)	0.400
Odorrana_schmackeri	4.788	8	0.885<br>(0.979)	0.693<br>(0.865)	7.376<br>(6.669)	6.472<br>(6.474)	4.214<br>(3.286)	3.983<br>(3.954)	35.502<br>(36.753)	30.420<br>(31.668)	0.500
Odorrana_tiannanensis	4.807	4	0.993<br>(0.897)	1.018<br>(0.433)	9.712<br>(1.981)	10.525<br>(4.090)	5.941<br>(2.539)	6.975<br>(0.596)	45.917<br>(15.377)	49.714<br>(27.645)	0.462

Occidozyga_lima	4.369	4	0.117 (0.102)	0.094 (0.136)	1.610 (0.632)	1.466 (0.870)	1.082 (0.400)	1.248 (0.355)	8.608 (9.237)	7.731 (8.762)	0.667
Ophryophryne_microstoma	4.464	3	0.310 (0.231)	0.299 (0.206)	2.137 (1.122)	2.111 (1.231)	0.967 (0.546)	0.985 (0.473)	9.146 (3.313)	8.825 (3.433)	1.000
Oreolalax_rugosus	4.722	1	0.386	0.322	3.782	3.072	1.280	1.359	17.470	15.874	0.877
Paa_boulengeri	4.855	2	0.733 (0.636)	0.853 (0.662)	9.983 (9.818)	11.008 (7.146)	5.421 (3.481)	5.402 (2.955)	56.54 (47.009)	55.655 (39.901)	0.500
Paa_robertingeri	4.889	2	0.863 (0.207)	0.888 (0.623)	9.562 (2.148)	9.926 (2.107)	5.206 (1.160)	5.601 (1.935)	51.412 (20.140)	53.578 (17.945)	0.429
Paa_sichuanensis	4.963	1	1.721	1.691	14.153	12.406	8.486	6.535	80.029	78.403	0.364
Paa_yunnanensis	4.962	4	1.694 (0.797)	1.885 (0.298)	17.979 (4.573)	20.636 (4.450)	9.052 (1.131)	10.115 (1.064)	96.171 (14.382)	110.262 (23.944)	0.421
Pelophylax_hubeiensis	4.734	8	0.596 (0.795)	0.535 (0.712)	3.245 (3.246)	3.230 (3.223)	3.174 (2.736)	3.263 (2.879)	29.320 (34.705)	25.763 (28.984)	0.857
Pelophylax_nigromaculatus	4.890	8	1.969 (1.443)	1.728 (1.280)	14.626 (8.421)	13.036 (7.170)	9.649 (4.260)	9.644 (3.883)	88.585 (38.243)	77.355 (39.837)	0.111
Pelophylax_pleuraden	4.780	8	1.286 (1.211)	1.275 (0.942)	8.851 (5.246)	8.998 (4.525)	5.177 (3.343)	5.117 (2.640)	45.957 (24.287)	44.476 (24.93)	0.133
Philautus_gracilipes	4.334	4	0.083 (0.057)	0.079 (0.045)	1.716 (0.342)	1.665 (0.185)	0.900 (0.073)	1.063 (0.147)	6.254 (7.175)	6.431 (0.909)	1.067

Polypedates_impresus	4.657	4	0.565<br>(0.280)	0.557<br>(0.407)	6.594<br>(1.926)	6.629<br>(1.397)	3.165<br>(1.455)	3.343<br>(0.624)	25.576<br>(8.698)	26.488<br>(26.147)	0.923
Polypedates_megacephalus	4.716	8	0.573<br>(0.395)	0.634<br>(0.702)	7.742<br>(1.717)	7.211<br>(2.693)	3.648<br>(1.609)	3.705<br>(2.041)	32.206<br>(13.813)	29.170<br>(0.395)	0.800
Polypedates_mutus	4.676	5	0.4521<br>(0.241)	0.489<br>(0.326)	5.920<br>(4.216)	5.847<br>(5.126)	3.008<br>(1.295)	3.055<br>(2.01)	23.692<br>(12.821)	22.067<br>(16.305)	1.200
Pseudorana_weiningensis	4.589	7	0.419<br>(0.315)	0.398<br>(0.322)	3.223<br>(1.036)	3.456<br>(1.479)	2.466<br>(0.343)	2.658<br>(0.664)	17.276<br>(15.581)	17.299<br>(15.32)	1.714
Rana_chaochiaoensis	4.751	8	1.010<br>(1.201)	0.965<br>(0.974)	6.116<br>(5.026)	5.692<br>(4.592)	3.619<br>(2.584)	3.908<br>(2.390)	34.820<br>(25.276)	32.068<br>(23.653)	0.667
Rana_chensinensis	4.735	5	1.003<br>(1.529)	0.879<br>(1.344)	4.912<br>(5.481)	4.186<br>(4.552)	3.114<br>(2.913)	3.027<br>(3.038)	28.668<br>(34.762)	28.642<br>(35.838)	0.286
Rana_hanluica	4.698	5	0.630<br>(0.686)	0.551<br>(0.333)	4.635<br>(1.254)	4.651<br>(0.680)	2.948<br>(0.438)	2.889<br>(1.312)	22.14<br>(4.005)	25.287<br>(4.796)	0.444
Rana_kukunoris	4.689	8	0.720<br>(8.621)	0.709<br>(0.392)	4.903<br>(2.525)	4.886<br>(2.588)	2.347<br>(0.812)	2.413<br>(0.792)	22.771<br>(10.312)	23.013<br>(8.926)	0.200
Rana_omeimontis	4.762	8	1.064<br>(0.635)	0.997<br>(0.471)	7.173<br>(2.730)	6.728<br>(2.090)	4.929<br>(1.531)	4.464<br>(1.351)	45.424<br>(12.862)	41.373<br>(13.870)	0.240
Rhacophorus_chenfui	4.587	2	0.173<br>(0.222)	0.181<br>(0.213)	3.278<br>(0.137)	2.726<br>(1.369)	1.933<br>(0.344)	2.509<br>(1.033)	13.952<br>(0.925)	15.899<br>(1.423)	1.333

Rhacophorus_dennysi	4.874	4	0.976<br>(0.646)	1.247<br>(0.677)	12.439<br>(5.010)	13.344<br>(6.045)	7.045<br>(1.758)	8.027<br>(3.840)	54.640<br>(28.970)	56.150<br>(26.232)	0.130
Rhacophorus_dugritei	4.678	8	0.477<br>(0.121)	0.503<br>(0.263)	6.104<br>(2.546)	5.706<br>(3.008)	2.689<br>(2.348)	3.030<br>(1.776)	22.249<br>(11.869)	23.047<br>(9.285)	0.308
Rhacophorus_feae	5.019	4	1.887<br>(0.780)	2.419<br>(0.939)	18.143<br>(4.502)	18.053<br>(3.141)	7.875<br>(1.923)	10.525<br>(1.495)	83.591<br>(14.748)	84.785<br>(21.77)	0.125
Rhacophorus_omeimontis	4.830	8	0.978<br>(1.008)	0.922<br>(0.768)	9.657<br>(5.897)	9.670<br>(5.631)	5.528<br>(2.974)	5.753<br>(3.039)	47.617<br>(32.518)	43.862<br>(23.121)	0.250
Scutiger_chintingensis	4.653	4	0.357<br>(0.258)	0.398<br>(0.117)	4.112<br>(1.390)	4.831<br>(1.850)	1.233<br>(0.461)	1.332<br>(0.472)	14.929<br>(3.599)	17.302<br>(3.359)	0.546
Xenophrys_leishanensis	4.525	2	0.209<br>(0.007)	0.206<br>(0.068)	2.053<br>(1.189)	1.901<br>(0.785)	0.661<br>(0.085)	0.940<br>(0.246)	6.965<br>(0.058)	7.137<br>(0.014)	1.250
Xenophrys_wushanensis	4.722	4	0.659<br>(0.130)	0.662<br>(0.215)	5.558<br>(3.589)	5.906<br>(4.504)	1.637<br>(0.645)	1.756<br>(1.113)	25.080<br>(11.497)	25.538<br>(13.089)	0.400

Table S3 PGLS models assessing the relationships between asymmetry index of total brain and three brain regions and the total brain size in species where the left hemisphere is smaller than the right one. Snout-vent length (SVL) was added as a covariate. Phylogenetic scaling parameters (superscripts following λ denote *P*-values of likelihood ratio tests against models with λ = 0 and 1, respectively).

Dependent variable	Brain regions	Independent variable	λ	R^2	*β*	*t*	*P*
Asymmetry index	Olfactory bulb	Total brain	$<0.001^{(<0.001,\ 0.462)}$	-0.004	0.009	0.274	0.786
		SVL			-0.040	-0.702	0.487
	Telencephalon	Total brain	$0.419^{(<0.001,\ 0.994)}$	0.035	-0.019	-1.763	0.086
		SVL			0.035	1.873	0.068
	Optic tecta	Total brain	$0.055^{(<0.001,\ 0.435)}$	0.017	-0.004	-0.298	0.766
		SVL			-0.009	-0.382	0.703
	Total brain	Total brain	$0.228^{(<0.001,\ 0.636)}$	-0.043	-0.004	-0.412	0.683
		SVL			0.009	0.548	0.587

Table S4 PGLS models assessing the relationships between the asymmetry index of different brain regions and predation risk in species where the left hemisphere is larger than the right hemisphere. Snout-vent length (SVL) was added as a covariate. Phylogenetic scaling parameters (superscripts following λ denote P-values of likelihood ratio tests against models with λ = 0 and 1, respectively).

Dependent variable	Brain regions	Independent variable	λ	R^2	*β*	*t*	*P*
Asymmetry index	Olfactory bulb	Predation risk	<0.001$^{(<0.001,\ 0.558)}$	0.015	0.006	0.974	0.335
		SVL			0.028	1.683	0.098
	Telencephalon	Predation risk	<0.001$^{(<0.001,\ 0.446)}$	0.080	-0.002	-0.586	0.561
		SVL			0.015	1.673	0.100
	Optic tecta	Predation risk	<0.001$^{(<0.001,\ <0.001)}$	0.130	-0.015	-1.901	0.071
		SVL			-0.002	-0.156	0.878
	Total brain	Predation risk	<0.001$^{(<0.001,\ 0.364)}$	0.088	-0.004	-1.553	0.126
		SVL			0.005	0.818	0.417

Table S5 The results of phylogenetic paired t-tests for the left and right hemisphere sizes in total brain and three brain regions (mean±SD) among 99 anuran species.

Brain regions	Left hemisphere (mm^3)	Right hemisphere (mm^3)	*t*	*df*	*P*
Olfactory bulb	2.719±0.398	2.712±0.396	0.575	96	0.566
Telencephalon	3.713±0.321	3.708±0.332	0.661	96	0.510
Optic tecta	3.678±0.364	3.397±0.357	-2.599	96	0.011
Total brain	4.357±0.352	4.348±0.358	0.704	96	0.483
